# Can reduce **-** the effects of chat-counseling and web-based self-help, web-based self-help alone and a waiting list control program on cannabis use in problematic cannabis users: a randomized controlled trial

**DOI:** 10.1186/1471-244X-13-305

**Published:** 2013-11-14

**Authors:** Michael P Schaub, Severin Haug, Andreas Wenger, Oliver Berg, Robin Sullivan, Thilo Beck, Lars Stark

**Affiliations:** 1 Swiss Research Institute for Public Health and Addiction at Zurich University, Konradstrasse 32, P. O. Box, 8031, Zurich, Switzerland; 2 Arud, Centres for Addiction Medicine, Konradstrasse 32, 8005, Zurich, Switzerland

**Keywords:** Cannabis, Internet, Chat, Web-based, Self-help, Cognitive behavioral therapy, Motivational interviewing

## Abstract

**Background:**

In European countries, including Switzerland, as well as in many states worldwide, cannabis is the most widely used psychoactive substance after alcohol and tobacco. Although approximately one in ten users develop serious problems of dependency, only a minority attends outpatient addiction counseling centers. The offer of a combined web-based self-help and chat counseling treatment could potentially also reach those users who hesitate to approach such treatment centers and help them to reduce their cannabis use.

**Methods/design:**

This paper presents the protocol for a three-armed randomized controlled trial that will test the effectiveness of a web-based self-help intervention in combination with, or independent of, tailored chat counseling compared to a waiting list in reducing or enabling the abstention from cannabis use in problematic users. The primary outcome will be the weekly quantity of cannabis used. Secondary outcome measures will include the number of days per week on which cannabis is used, the severity of cannabis use disorder, the severity of cannabis dependence, cannabis withdrawal symptoms, cannabis craving, the use of alcohol, tobacco, and other non-cannabis illicit drugs, changes in mental health symptoms, and treatment retention. The self-help intervention will consist of 8 modules designed to reduce cannabis use based on the principles of motivational interviewing, self-control practices, and methods of cognitive behavioral therapy. The two additional individual chat-counseling sessions in the additional chat condition will be based on the same therapy approaches and tailored to participants’ self-help information data and personal problems. The predictive validity of participants’ baseline characteristics on treatment retention and outcomes will be explored.

**Discussion:**

To the best of our knowledge, this will be the first randomized controlled trial to test the effectiveness of online self-help therapy in combination or without chat counseling in reducing or enabling the abstention from cannabis use. It will also investigate predictors of outcome and retention for these interventions. This trial is registered at Current Controlled Trials and is traceable as ISRCTN59948178.

## Background

Cannabis is the most commonly used illicit drug in the developed world; e.g., a rough European estimate suggests that approximately 22% of Europeans within the ages of 15 and 64 have tried cannabis, and approximately 6.8% of this population report using cannabis in the past month. This represents 12 million Europeans, of whom 25% report daily cannabis use [1]. Switzerland has the third highest national prevalence of cannabis use in Europe; the lifetime prevalence rate is 27.9% (men 32.8%, women 23.2%), and the 12-month prevalence rate is 5.1% (men 7.5%, women 2.8%) [2]. The age group with the highest prevalence is between 15 and 24 years; this group has a 17.3% 12-month prevalence rate, and among these users nearly one fifth use cannabis daily [2].

Despite these high numbers, only a minority of cannabis users is at risk of cannabis dependence. Currently, the estimates of the risks of cannabis dependence based on general population surveys suggest that between 10 and 11 percent of all cannabis users develop cannabis dependence [3,4]. However, the risks of cannabis dependence [5] and cannabis use problems [6] are much higher among cannabis users who consumed cannabis in early life. Daily cannabis use is associated with poorer mental and physical health, lower educational achievement, and decreased cognitive functioning [7]. Moreover, there is clear evidence from numerous studies that frequent cannabis use is strongly associated with a broad range of primary mental illnesses [8]. Thus, specific attention should be paid to mental disorders in heavy cannabis users.

Treatment demand statistics from Swiss outpatient addiction treatment centers demonstrate a linear increase in new treatment entry cases in which cannabis is main problem substance from 2003 (12%) to 2011 (46%) [9]. The main group of cannabis use disorder treatment seekers consists of young males between the ages of 15 and 24 [9]. In Europe, an increase in the demand for treatment in which cannabis is the main problem substance has also observed, although smaller than that in Switzerland [10]. However, it is obvious that the majority of problematic cannabis users, of whom it is estimated that one-half develop cannabis dependence [5] and many demonstrate co-occurring mental health problems, is still not in treatment.

There are several explanations for the low treatment attendance rate of problematic cannabis users: 1) accessibility—in many European countries and especially in rural locations, addiction treatment services are scarce, or treatment seekers are required to travel long distances to more urban areas for access to treatment. Moreover, the operating hours of addiction counseling centers are typically during office hours, which make attendance difficult for those cannabis users who have jobs and work during these hours; 2) stigmatization and need for social distance—many problematic cannabis users do not want to be stigmatized, desire personal distance from the counselor [11], or simply want to quit on their own [12]; and 3) the scarcity of insight into the problem affects motivation to change—many users see the risk of the development of minor mental health problems, such as mild depression symptoms, during ongoing problem cannabis use but rarely see the physical risks such as the development of COPD, lung cancer, and heart disease; this phenomenon is more apparent in frequent users than occasional users [13,14]. Those who are aware of such physical risks from problematic cannabis use are also more ready to quit [13]. Raising awareness of cannabis-related physical health risks might also increase intentions to reduce or quit cannabis use. In general, the principle of concurrent cover (i.e., non-invasive, low-cost interventions in which therapeutic intensity can be enhanced according to need) appears to be an appropriate mean for problematic cannabis users to lower their ever-increasing health care costs [15], and this consideration is of interest to Switzerland and other industrialized countries suffering from exorbitant health costs.

Web-based self-help programs that reduce problematic consumption are able to facilitate access to consumer groups in the general population that do not want to be stigmatized. Such programs have consistently been shown to be effective in the reduction of problematic alcohol use [16,17] and (although less efficiently and consistently [17]) in tobacco smoking cessation [18,19]. Interestingly, the availability of web-based self-help with individual chat sessions has been demonstrated to aid the reduction of alcohol use and alcohol disorder-related symptoms in problematic alcohol users [20].

Studies of web-based programs for the reduction of the most frequently used illicit drugs, such as cannabis, cocaine and amphetamines, are scarce [21,22]. Currently, there are only two specific web-based programs for the reduction of cannabis use that have been investigated in randomized controlled trials, and these programs employ different intervention approaches [23,24]. The German “Quit the Shit” program [24] is based on principles of self-regulation and self-control and is a solution-focused approach. This program is structured into weekly personalized feedback sessions based on participants’ consumption diary entries, intake and termination chats, and the total allowed program time is 50 days. Attrition in the German study of Tossmann and colleagues [24] was high and higher in the intervention (11.6%) condition than in the waiting list control condition (24.7%). Nevertheless, these authors found significant effects on cannabis use reduction in their per-protocol and last observation carried forward analyses. The Australian program “Reduce Your Use: How to Break the Cannabis Habit” [23] is a fully automated self-help intervention consisting of 6 modules for the amelioration of cannabis use disorders based on cognitive-behavioral therapy (CBT) approaches [25,26], motivational interviewing (MI [27]), and behavioral self-management (BSM [28]). This program was tested for effectiveness in a randomized-controlled trial and compared to a psycho-educative control condition also consisting of 6 modules. Study retention was higher in the intervention and the control condition after 6 weeks (66% vs. 64%) and at a 3-month follow up (54% vs. 52%) than in the aforementioned German study [24]. The frequency of cannabis use and the quantity of cannabis consumed were both more reduced in the intervention group than in the control group at 6 weeks and at the 3-month follow up.

The combination of a fully automated self-help intervention based on therapy approaches of the study of Rooke and colleagues [23] with additional individual chat sessions for the reduction of cannabis use could potentially increase the effectiveness of interventions for problematic cannabis as has been demonstrated for the reduction of alcohol use in problematic alcohol users [20]. More interactive web-based interventions may be more effective in reducing cannabis use in problematic cannabis users. However, the results of the Australian study [23] need confirmation by other studies. Specifically, the high rates of study retention in the intervention and control groups may be due to limited geographic access of many rural users to the addiction treatment system in Australia, which did not appear to be the case in the German study, which encountered considerable retention problems [20]. Therefore, the treatment approaches adopted for web-based self-help by Rooke and colleagues [23] should also be validated in countries with high densities of addiction counseling services such as Switzerland.

The current study aims at to investigate and compare the effectiveness of web-based self-help interventions in combination with tailored chat counseling based on CBT, MI, and BSM in reducing cannabis use in problematic cannabis users. More specifically, this study aims to compare the effectiveness of interventions that include chat-counseling and web-based self-help, web-based self-help alone and a waiting list control condition in the reduction of cannabis use in problematic cannabis users in a three-arm randomized controlled trial.

### Study interventions

**Figure 1:**
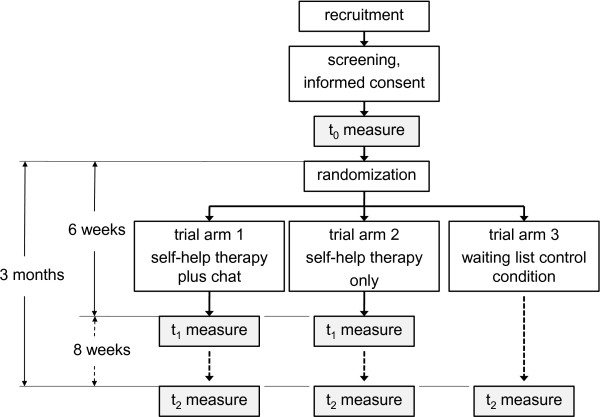
**Trial flowchart.** This figure provides an overview of the participant flow for this trial.

The intervention in the first arm consists of the web-based self-help intervention from study arm two in combination with two individual chat-counseling sessions based on MI and CBT approaches that are tailored to the data the participants entered into the self-help intervention and individual requests. The web-based self-help intervention “Can Reduce” from study arm two is based on classical CBT approaches for the treatment of cannabis dependence [26], MI approaches [27], and BSM [28]. Study arm three consists of a classical waiting list. Figure 1 presents a detailed overview of the aforementioned study arms.

Study arm one and two involve weekly reminder e-mails to login and fill out a consumption diary. The following modules, organized into three main parts, are offered as a web-based self-help intervention (study arms one and two):

• Part 1: Introduction

• Registration process

• Explanation of the cannabis consumption diary and its fully automated progress charts and statistics

• Examination of the pros and cons resulting from a change in cannabis consumption patterns and further principles of motivational interviewing to address motivation

• Explanation of the “My Can Reduce” folder (this folder allows individuals to review the acquired summarized module documents, e.g., the list of the top five strategies for dealing with cannabis cravings)

• Explanation of the emergency button for immediate responses to frequently asked questions and access to emergency contacts

• Part 2: Key Modules (participants are encouraged to complete these modules in the order presented below)

• Module 1: Strategies for goal achievement

• Module 2: Identifying risk situations

• Module 3: Dealing with cannabis craving

• Module 4: Dealing with relapses

• Part 3: Further Modules (participants are encouraged to complete at least two, in any order)

• Module 5: Tobacco smoking during the reduction of cannabis use

• Module 6: Saying “no” to foster refusal skills

• Module 7: Dealing with burdens

• Module 8: Preserving achievements

Furthermore, a glossary that explains the terms, definitions, and concepts used in the intervention, information about the history of cannabis use, the short-, medium- and long-term effects of cannabis, the physical risks of cannabis (COPD, lung cancer, cardio-vascular, etc.), co-occurring mental health problems (depression, psychosis, ADHD, etc.), and harm reduction techniques for the use of cannabis with recommendations (these recommendations favor oral intake and are based on the recommendations for medical cannabis use [29,30]) will be provided in an appendix accessible after login, along with frequently asked questions and their corresponding answers.

The additional two chat counseling sessions in study arm one will apply support regarding behavioral change according to MI, discuss the modules of the web-based self-help part based on MI and CBT, review the development of the consumption diary and will be structured as follows:

• Chat session 1: Starting point and objective agreement

• Personalized feedback according to baseline assessment of cannabis related physical and mental health risks

• Review of successful strategies of change related to topics other than cannabis

• Imagining daily routines after cannabis cessation; exploration of aims to live without, and the dissonances of, cannabis use; mobilization of social support with the invitation for a relevant third person to join chat session 2

• Agreeing on a cannabis use objective expressed in tangible numbers (e.g., a maximum of two standard joints per week, complete cannabis abstinence etc.)

• Chat session 2: Experience exchange and consolidation

• Experience exchange regarding the agreed-upon objective since the starting point

• Review of the patterns of the consumption diary goals and achievements

• Review and consolidation of web-based self-help modules

• Experience and improvement of social support

• Revising objectives

• Saving strategies for long-term success

The web-based self-help intervention and the subsequent tailored chat counseling aim to reduce cannabis use. However, those participants who seek cannabis abstinence will also be encouraged to make step-by-step reductions until full abstinence has been reached. The maintenance of abstinence from one day to the next, as is recommended by the content of tobacco prevention websites [31], will not be advised for safety reasons (e.g. to avoid possible severe withdrawal symptoms).

Participants randomized to the waiting list will have the opportunity to participate in the web-based self-help intervention 3 month after registration. Follow up measures for those participants will be assessed online or by telephone interviews for those participants who cannot be reached online at the 3 month follow up.

## Methods/design

### Aims of the trial

This study aims to compare the effectiveness of a web-based self-help intervention alone or combined with chat counseling in the reduction of the cannabis use of problematic cannabis users via a three-arm randomized controlled trial. The primary outcome is the weekly quantity of cannabis consumed (measured in the number of standard joints per week) at the 3-month follow up. The secondary outcome measures include the frequency of cannabis use days (7 day point prevalence of cannabis use days), changes in problematic cannabis use, severity of cannabis dependence, cannabis withdrawal symptoms, cannabis craving, the use of other psychoactive substances, mental health symptoms, functional health states, intervention satisfaction, and treatment retention. The predictive validity of participant characteristics for treatment retention and primary and secondary outcomes will be explored.

### Study population

The study population will be recruited through the Can Reduce website, several websites from local outpatient treatment centers, and from nightlife prevention websites. In addition, advertisements will be placed in internet-forums and (commuter) newspapers.

### Hypotheses

We hypothesize that web-based interventions, which are more interactive, will be more effective in reducing cannabis use among problematic cannabis users. We will test the following detailed study hypotheses with respect to the main outcome, i.e., reduction of the quantity of cannabis used between the baseline and the 3-month follow up:

1. Tailored chat-based counseling in combination with web-based self-help for the reduction of cannabis use (study arm 1) is more effective than the waiting list control condition (study arm 3).

2. Web-based self-help for the reduction of cannabis use (study arm 2) is more effective than the waiting list control condition (study arm 3).

3. Chat-based counseling in addition to web-based self-help for the reduction of cannabis use (study arm 1) is by trend more effective than web-based self-help alone (study arm 2).

Moreover, we expect the participants of study arm 1 to improve more than those in study arm 2 with respect to the secondary outcomes between the baseline and the three-month follow up termination.

### Measurement instruments

The primary outcome measures of cannabis consumption will be recorded as the 7-day point prevalence of the quantity of cannabis used, quantified in pre-defined standard cannabis joint sizes, as specified in the consumption diary (see also Table 1). In the first step, participants will choose between three different cannabis forms presented in photographs (low potency cannabis plant, high potency cannabis plant, or cannabis resin). In the second step, five different standard joints for every category will be presented (1/10 g, 1/6 g, 1/4 g, 1/3 g, 1/2 g; pictures will come from the global drug survey cannabis meter [32]); these joints will either be pure cannabis or cannabis mixed with tobacco. A standard tobacco cigarette, a ruler with centimeter and millimeter scales, the fraction amount in grams, and an open 10 cm paper prepared to roll a joint and containing the cannabis plant/resin-tobacco mixture or pure cannabis will be presented. Participants will choose which picture most closely approximates the cannabis joints they most often smoke. Those who smoke cannabis in different preparations (e.g., in a bong, a vaporizer, etc.) will choose the photograph that most closely approximates the amount of cannabis their usually consume. The final chosen picture will be placed in the individual consumption diary, and participants will be asked to convert the quantities of the cannabis they smoke into units relative to that picture if they consume cannabis for once in forms other than their common standard joint (rule of thumb that one high potency joint is equal to two low potency cannabis joints of identical content weight, one cannabis resin joint is equal to three low potency joints of identical content weight, etc.; identical content weight pictures will be presented on a separate page). Participants will also have the opportunity to indicate quantities below one common standard joint down to one tenth of a joint (they will also be able to indicate quarter-joint). Quantities smaller than one joint are expected for those who share cannabis joints with others.

**Table 1:** Measurements and instruments

**Assessments/instruments**	**Baseline**	**1 week**	**3 weeks**	**6 weeks**	**3-month follow up**
Socio-demographics	x				
MHI-5	x				x
CLAMES^1)^	x				x
Quantity of Cannabis use^2)^	x	x	x	x	x
Frequency of Cannabis use^2)^	x	x	x	x	x
CUDIT	x				x
SDS	x			x	x
CWS		x	x		
CCS-7		x	x		
FDA	x			x	x
Intervention Satisfaction				x	

^1)^ Additional retrospective cost-relevant information directly or indirectly related to cannabis use will be collected by the CLAMES to conduct cost-effectiveness and cost-utility analyses.

^2)^ 7-day point prevalences of the quantity (in common standard joints) and frequency (the number of days on which cannabis is used) of cannabis use will be derived from the consumption diary assessed daily using data from the last 7 days.

Moreover, the following secondary outcome instruments will be applied: 1) cannabis consumption days per week according to the consumption diary; 2) the Cannabis Use Disorders Identification Test (CUDIT), which is a 10-item questionnaire [33] that was constructed by adapting the Alcohol Use Disorders Identification Test (AUDIT [34]). To cover the length of the trial length, this instrument will be adapted to focus on the last 3 months in its planned assessments (baseline and 3-month follow up); 3) The Severity of Dependence Scale (SDS), which is a 5-item questionnaire that measures the severity of cannabis dependence. Each of the five items is scored on a 4-point scale (0–3). The total score is obtained through the addition of the ratings on all 5 items. High scores indicate high levels of dependency [35]; 4) The Cannabis Withdrawal Scale (CWS) [36], which is a 26-item questionnaire containing statements that describe cannabis withdrawal symptoms within the last 24 hours on 11-point scales (0–10); 5) The Cannabis Craving Symptoms questionnaire (CCS-7), which is a 7-item questionnaire [37] derived from the Marijuana Craving Questionnaire [38]. Each item is rated on a 7-point-scale (1–7); 6) the “Fragebogen Substanzanamnese” (FDA), which is a questionnaire that ascertains the number of years of consumption over the lifetime, the past month’s consumption, and the manner of consumption for DSM-IV/ICD-10 substances of abuse. This measure was derived from the Europe ASI [39]; 7) the short version of the Mental Health Inventory (MHI-5) [40], which is a validated and user-friendly self-assessment questionnaire that assesses recent mental distress and self-reported diagnoses of depression; 8) the CLAMES, which is a standardized measure of functional health states that provides a simple, generic measure of health for clinical and economic appraisal. This instrument consists of a descriptive system to measure pain/discomfort, physical functioning, emotional state, fatigue, memory and thinking, social relationships, anxiety, speech, hearing, vision, and use of hands and fingers on four or five levels that range from no problems to extreme problems [41]. Overall intervention satisfaction will be ascertained on a visual analogue scale from absolutely unsatisfied to absolutely satisfied. Finally, retention will be derived from the last cannabis use input entered into the consumption diary in study arms one and two.

### Estimation of the expected effect sizes and sample size

Based on results of the study of Rooke and colleagues [23], we expect small to medium effect sizes for the reduction of the quantity of cannabis used and the frequency of cannabis use between study arm 2 (web-based self-help without chat counseling) and study arm 3 (waiting list control) of at least 0.15 (Cohen’s f) between t0 and t2. Assuming that the study arm 1 (web-based self-help with chat counseling) will result in a larger Cohen’s f, we rely on the aforementioned comparison (arm 2 vs. arm 3) for sample size estimation. A sample size of n = 89 in each study group would have 80% power (F-test, α = 5%) to detect this difference based calculations performed with the G-Power software. Thus, we aim to recruit a total of 267 participants.

### Consent procedure

The rationale of the study will be explained to the participants. The participants will then be informed about the following: (1) study inclusion and exclusion criteria (see Table 2); (2) the three different arms and their 1:3 chance of being allocated into one of the arms; (3) the potential risks of participation; (4) safety arrangements during and after the study phase; (5) the inability of Can Reduce (with or without chat counseling) to replace face-to-face therapy for problematic cannabis use/abuse; and (6) the circumstances under which they should contact their general practitioner or a professional from a medical advisory and emergency list that will be accessible at all times via an emergency button. The participants will also be informed that the study has been reviewed by the ethics committee of the Canton of Zurich and given a declaration of no objection (nihil obstat). Moreover, they will be informed about their right to withdraw from the study at any time without consequences except for the loss of further compensation. Informed consent will be accepted when participants click on a field on the informed consent page and submit the consent by clicking a submission button.

**Table 2:** Inclusion and exclusion criteria and rationale

***Inclusion Criteria***	**Reasoning**
- Minimum age of 18 years	To ensure a minimal age of participation
- At least once a week cannabis use over the 30 days prior to study entry	To include occasional users to provide extended study validity
- Current serious psychiatric disease or history of psychosis, schizophrenia, bipolar type I disorder or significant current suicidal or homicidal thoughts	To avoid exacerbation of serious symptoms of these severe psychiatric diseases
- Other pharmacological or psycho-social treatments for cannabis use disorders	To avoid confounding treatment effects
- Inability to read or write in German	To avoid confounding drug effects
- For women: pregnancy and breastfeeding	To prevent subjects with these conditions from entering the study

### Baseline assessment

After providing informed consent, subjects who meet study entry criteria will create a personal and secure login and password (with automated real-time verification of the passwords’ security level) and will receive an automated e-mail notification with their access information. They will then be directed to the baseline assessment of socio-demographic characteristics and consumption patterns (see Table 1). Participants that do not meet the inclusion and/or meet one of the exclusion criteria (see Table 2) will receive an explanation of why they will not be permitted to participate in the study and will receive further recommendations (e.g., not to reduce their consumption of cannabis before visiting a physician and receiving any necessary treatment, etc.). A decision tree for the possible inclusion and exclusion criteria combinations will be constructed and implemented. The completion of the baseline assessment will allow participants to begin their study arm according to an automated online allocation procedure. Participants that do not fulfill the criteria can proceed with the modules from the Can Reduce web-based self-help intervention, but they will not participant in the study (i.e., there will be no assessments or compensation).

### Randomization and allocation

Once participants have completed their baseline assessment, they will be randomized by a computer program in a 1:1:1 ratio to 1 of 3 “parallel” groups, and this assignment will be automatically registered in the background database. As we offer full transparency on the three groups, we expect a certain risk that some might try to circumvent their assignment by registering another account in hope to end up in a different group. In case a participant surmounts the administrative hurdle, he nevertheless will be assigned to the same group for a certain amount of time, based on his IP-address.

### Safety

During the 6-week therapy/intervention phase, participants will have the opportunity to contact an outpatient clinic in a nearby city by telephone (lists with hours of operation, web-links, postal addresses, and telephone numbers will be provided in the appropriate language). Additionally, a medical advisory and emergency list that is based on the web-based treatment guidelines of the Federation of Swiss Psychologists [42] (in line with the HONcode [43], a code of ethics for medical information on the Internet) will be provided for cases of emergency. This list will always be accessible to participants in all study arms before, during, and after study participation regardless of whether the participants withdraw from the study. This list will include phone numbers for emergency help lines and the contact information of the study team and the webmaster.

### Experience, training and supervision of chat counselors

To ensure that chat counseling sessions in study arm one are conducted in the same MI style and cover all relevant points, the chat counselors will be trained MI counselors with at least one year of experience in treating cannabis-abusing patients who will follow a check list in each counseling session. Two senior psychiatrists with extended face-to-face and web-based addiction counseling/therapy experience will train and supervise junior counselors. Specific addiction chat counseling quality standards will be developed and implemented for this study based on the EQUS treatment quality standards [44] and extended to Swiss national addiction counseling quality standards.

### Trial flow

Figure 1 provides an overview of the trial flow. If a participant successfully completes the baseline assessment (t0), he or she will be introduced step-by-step into the corresponding study arm, invited to participate in part one (study arms 1 and 2), or informed that they will be provided access to the web-based self-help within approximately 12 weeks (waiting list condition). Participant in the intervention arms 1 and 2 will receive automated e-mail notifications to login and enter their cannabis consumption frequency and quantity into their consumption diary every week. Access to all modules will be unrestricted from day one. However, the participants will be encouraged to complete all key modules (modules 1–4) in order, to complete at least two further modules chosen from modules 5 to 8 and to repeat any modules they feel they need or that they perceived as helpful within the 6 weeks of duration of study arms 1 and 2.

Six weeks after the baseline assessment, participants will again be invited by e-mail to login and complete the final study assessment (t1). Participants will be invited to complete the follow up assessment (t2) by an e-mail message that will be sent 3 months after t0; this message will inform the participants that completion of the entire 3-month follow up assessment will be compensated with 40 Euro (via an online voucher or an online charitable donation).

### Handling of study dropouts

Each week, participants in intervention arms one and two will be sent an automated e-mail that will contain a reminder to login to their consumption diary, a reminder to work on their modules and a direct link to the Can Reduce login site. If participants do not log in, they will receive a reminder e-mail every three days for the following two weeks. If they do not continue their participation after these reminders, they will be considered to have dropped out of the study. Participants allocated to study arm 1 (chat and self-help) will additionally be invited to the first chat session at least three times over two weeks if they do not respond. In each of these invitations they will be asked to agree to attend two chat sessions on dates suggested by their counselor. If the participants are unable to attend on these dates, they will have the opportunity to suggestion dates to the counselor. This procedure will be repeated until a first chat date has been found. If participants do not show up at the agreed chat date they will be sent new chat dates until a first chat appointment occurs. If participants do not reply after three invitations to schedule chat appointment, they will be considered to have dropped out of the study. The second chat appointment will be scheduled at the end of the first chat session. If participants do not show up for the second session, they will be sent new chat dates twice. Attendance of at least one chat appointment will be required of the participants, otherwise they will be considered to have dropped out. Participants in study arm 1 who discontinue their consumption dairies but proceed with the chat session will not be considered as drop outs and will be included in the study.

### Data analysis

Data will be analyzed according to the intention-to-treat principle (ITT). For the ITT analyses, we will apply the multiple imputations procedure (MICE) of STATA, which imputes missing data using all available baseline variables (sociodemographic and health- and cannabis-related variables). Baseline measurements will be compared using t- and Chi-squared tests. Differences between primary and secondary outcome variables between baseline and the follow up points will be tested using generalized estimating equation (GEE) models. GEE is a repeated-measures regression model that takes into account the correlations between the repeated measures from each person [45]. We will perform logistic GEE analyses for the binary outcome variables and linear GEE analysis for continuous outcome variables. Results from the imputed data set will be cross-checked with the non-imputed data set.

We will conduct additional exploratory regression analyses to determine whether baseline variables predict the severity of cannabis use disorder (CUDIT, SDS), cannabis craving (CCS-7), cannabis withdrawal (CWS), reduced psychiatric symptoms (MHI-5), and treatment retention. For these analyses, we will use linear, multinomial, or binary regression models depending on the scale of the outcome measure.

### Ethical review

This RCT will be executed in compliance with the Helsinki Declaration and has been reviewed by the ethics committee of the Canton of Zurich who have given their declaration of no objection (KEK-StV-Nr. 15/13).

## Discussion

To the best of our knowledge, this will be the first randomized controlled trial to test the effectiveness of online self-help therapy alone or in combination with chat counseling in reducing or enabling the abstention from cannabis use. This study will add to the ongoing discussion of whether web-based interventions that are more interactive are more effective in reducing substance use in problematic users [20]. This study will also be the first to investigate predictors of outcome and retention for online self-help and for chat counseling that aim to facilitate the reduction or cessation of cannabis use. If the efficacy of this form of self-help is demonstrated in this RCT, this self-help intervention will be integrated into the basic services for cannabis users of the national addiction internet portal of Switzerland and will potentially be able to reach those cannabis users who have difficulties attending outpatient addiction treatments that follow the principle of concurrent cover [15]. Specifically, people who rely on evening sessions after work, those in need of social distance, and those worrying about stigmatization [11] may profit from this new addiction intervention. This intervention will foster problem insight regarding physical problems that can accompany ongoing problematic cannabis use [13,14] utilizing a non-directive MI self-help intervention and/or chat counseling style.

Previous face-to-face therapy intervention research has demonstrated that the goal of cannabis abstinence and the desired goals regarding the reduction of cannabis use change frequently in problematic cannabis users [46], especially in those who choose abstinence as the agreed-upon aim of therapy. Failure to reach the fixed consumption aim might result in frustration, self-deception, or in concealment of cannabis relapse from the therapist during face-to-face therapy, which may result in misleading effects. In contrast to face-to-face therapy, Can Reduce is anonymous and asks participants to re-login weekly and to set daily consumption aims week-by-week after the evaluation of past cannabis use. Moreover, the chat counseling sessions are tailored to the aims reported in the consumption diary and reported use. Thus, we expect self-deception and concealment to be less pronounced in the intervention arms.

Some participants may want to reduce their cannabis use only marginally and might reach their aims in the first weeks of the intervention, which might result in discontinuation of participation in the online self-help intervention. Therefore, we will recommend that participants reduce their cannabis by at least 20 to 30 percent in the first week of the intervention and continue with this strategy in subsequent weeks if they succeed. For participants who do not succeed, we will recommend that they create more modest goals until their final aim is received. Participants receiving chat counseling will additionally be motivated by their counselors to avoid setting their goals too low.

During the online implementation of the consumption diary of the web-based self-help intervention, pilot participants stressed the importance of the definition of common standard joints as the most appropriate measure for the consumption diary. On the one hand, the established cannabis meter pictures from the global drug survey website facilitated the pilot participants’ entry of their cannabis use, and, on the other hand, they felt more motivated by a tailored photograph than a written instruction. In our opinion, this is the most effective method to collect appropriate quantity measures, especially from cannabis users who use cannabis several times a day. However, as this is the first time this kind of quantity measure will be used in a randomized controlled trial, we will also rely on the number of days per week that cannabis is used.

One specific problem involves the online implementation of informed consent. As in previous research [21], we will rely on participants to click on a field on the informed consent page and submit the consent with a submission button. Thus, we must trust that the participants have read and understood the study information and that they are at least 18 years old. However, as we followed the recommendations of [47] and [48] regarding the best implantation of online informed consent procedures, we expect that the participants will read the study information in full and provide informed consent.

Finally, another potential problem for this trial is the expected high attrition rates at the end of online therapy and at the follow up; this attrition will also likely be higher in the waiting list control group. We will address this issue in three ways: 1) all participants must invest approximately 20 minutes for the baseline assessment, which will select more motivated participants and prevent the participation of un-motivated participants; 2) participation in the 3-month follow up assessment will be compensated by a 40 Euro incentive (an online voucher or an online charitable donation); 3) all missing values in the final data set will be imputed, a promising approach that, as shown by our Dutch colleagues, has become increasingly important in e-health research [49].

## Competing interests

The authors declare that they have no competing interests. This trial is registered at Current Controlled Trials and traceable as ISRCTN59948178.

## Authors’ contributions

MPS was responsible for the study design, prepared the first draft of the paper and the final manuscript. MPS, SH, OB, AW, RS, TB and LS developed the intervention of study arms one and two. AW programmed and implemented the study website Can Reduce. All authors read and approved the final manuscript.

## Pre-publication history

The pre-publication history for this paper can be accessed here:

http://www.biomedcentral.com/1471-244X/13/305/prepub
